# Effect of Graphite Aging on Its Wetting Properties and Surface Blocking by Gaseous Nanodomains

**DOI:** 10.1021/acs.langmuir.3c02151

**Published:** 2023-09-21

**Authors:** Hana Tarábková, Pavel Janda

**Affiliations:** Department of Electrochemical Materials, J. Heyrovský Institute of Physical Chemistry, Czech Academy of Sciences, Dolejškova 2155/3, CZ-182 23 Prague 8, Czech Republic

## Abstract

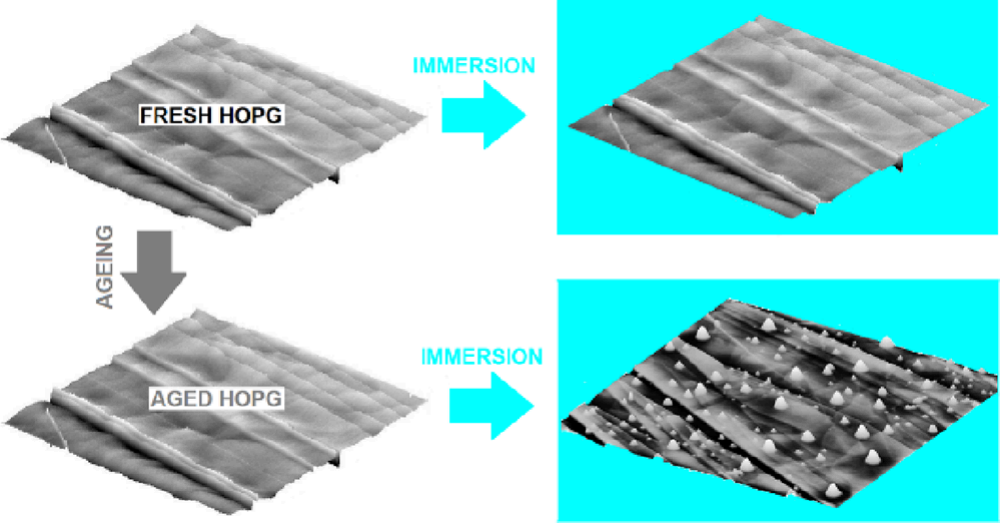

Early works considered basal planes of highly ordered pyrolytic graphite (HOPG) as hydrophobic, relatively inert materials with low electrocatalytic activity due to nonpolar sp^2^ carbon. On the contrary, a freshly prepared HOPG surface exhibits intrinsically mildly hydrophilic properties, with a low contact angle of water, which increases after exposure to an ambient atmosphere. This process, called aging, ascribed to adsorption of airborne hydrocarbons, is reportedly accompanied by strong decay of electron transfer kinetics, the mechanism of which is not yet fully understood. Examining both freshly prepared and aged basal plane HOPG immersed in water by PeakForce quantitative nanomechanical imaging, we have found that aged HOPG is occupied by ambient gaseous nanodomains, the existence of which is explained by incomplete wetting. They cover up to 60% of the immersed surface and their incidence is in direct relation with graphite aging time. In contrast with aged graphite, gaseous nanodomains were absent on the freshly stripped HOPG surface. It can be concluded that ambient gaseous nanodomains can prevent aged basal plane HOPG from contact with aqueous media and may thus affect processes at the solid–liquid interface.

## Introduction

Highly oriented pyrolytic graphite (HOPG) represents an important material composed of sp^2^ hybridized carbon atoms in a honeycomb (graphene) arrangement, where multiple highly ordered graphene layers (basal planes) are stacked to make a well-defined structure. Its basal plane with terraces and step edges is easily characterized by a variety of techniques and, similar to other sp^2^ bonded allotropes, has drawn substantial attention as a supporting material for heterogeneous catalysis, manufacturing electrodes for electrochemistry, sensors, etc.^1,2^ As the HOPG basal planes can be cleaned efficiently by simple peeling-off of the topmost layers, yielding a well-defined surface, a full exploration of its electrochemical properties under controllable conditions represents a useful benchmark from which the intrinsic properties of graphene can be understood as well. Accordingly, the results obtained on HOPG can be, with good approximation, utilized to elucidate the behavior of other carbonaceous graphene-based surfaces.^3^ As the basal plane HOPG is frequently utilized as an electrode in electrochemical processes, it is important to clarify its property and behavior in contact with liquids and with aqueous media in particular. The quality of this contact and composition of the graphite–water interface affect the reliability of kinetic studies of processes taking place at the solid–liquid interface.

Early works regarded the HOPG basal plane as a hydrophobic,^4^ relatively chemically inert material with very low electrocatalytic activity, even for outer-sphere redox reactions. Its hydrophobicity was assumed to be due to the sp^2^ carbon, which is nonpolar. But, recent studies^5−7^ have shown that the graphite surface is in fact intrinsically mildly hydrophilic with surprisingly high charge transfer activity of basal plane. Liu et al.^8^ found a relatively low contact angle of water (CAW) on freshly prepared supported graphene and graphite surfaces, indicating their hydrophilicity. The value of CAW, however, increases after surface exposure to an ambient atmosphere due to so-called “aging”. The mechanism of this process is ascribed to adsorption of airborne hydrocarbons,^4^ which can be removed by thermal annealing or by UV ozone treatment. Dryfe et al.^9^ found declining capacitances of HOPG in the order of freshly exfoliated - aged in an inert atmosphere - aged in air, while CAW was rising in the same direction, indicating that the HOPG surface was modified by exposure to air. Studies utilizing low voltage SEM^10^ and force curve analysis^5^ revealed formation of unevenly distributed spots ascribed to contaminant adlayers. The presence of ambient gas on a water-immersed HOPG surface was predicted by the MD simulation, which indicates enrichment of liquid phase by dissolved ambient gas near immersed HOPG/SLG/MLG surfaces^11^ more than 2 orders of magnitude, compared to bulk liquid, though no relation to aging or to the immersion process was stated. The exact mechanism of immersion by which a liquid front advances across a solid surface (i.e., the wetting process) remains not yet fully understood^12^ though it determines the quality of contact between the solid and liquid phase, which in turn affects efficiency of all processes taking place at the solid–liquid interface. Wastl et al.^13^ reported on solid monolayers of aggregated ambient gas molecules (air) adsorbed on a hydrophobic surface. Similarly, Hwang et al.^14^ ascribed a row-like ordered nanostructure observed at a graphite–water interface to adsorption of nitrogen molecules and gas segregation in adlayers, though the identical nanostructure was observed also *ex situ* on dry HOPG basal plane surfaces^15^ and identified by TEM as graphene-based nanostructures. Kinetic studies performed on HOPG electrodes in aqueous media reveal strong correlation of electrochemical rate constants with HOPG aging, which was found to slow down the reaction kinetic.^16−18^ The contamination (“aging”) by airborne contaminants is known to decrease also the electrocatalytic activity of catalysts like Pt and Pd.^19^ Though gaseous composition of nanobubbles may influence their shape by pinning and contact angle as reported, e.g., Hu et al.,^20^ pure atmospheric gases themselves are not responsible for the contamination effect. While numerous studies^21−29^ report on gaseous nanodomains residing on a water-immersed HOPG surface, papers correlating their incidence with surface aging are scarce.^30^

This paper investigates the relation between aging of basal plane HOPG exposed to an ambient atmosphere and the existence of surface gaseous nanodomains explained by incomplete wetting.

## Materials and Methods

An atomic force microscope Dimension Icon (Bruker, USA) was utilized for topography imaging, PeakForce quantitative nanomechanical analysis (PFQNM), and tapping mode (TM) phase imaging, all performed in a setup for scanning in liquids. Cantilever SCANASYST-FLUID (resonant frequency *f* = 150 kHz, spring constant *k* = 0.7 N/m, nominal tip radius 20 nm, Bruker, USA) with an intentionally dull tip for scanning in fluids was utilized both for PFQNM and tapping *in situ* after hydrophilization by UV irradiation (254 nm/10 W (3.3 mW/cm^2^), PenRay Lamp, UVP Analytik Jena GmbH, Germany) for 5 h. The hydrophilization of the AFM tip prevents its penetration via the gas–water interface of the gaseous nanodomain into the gas phase (which has hydrophobic properties) and to disturb its scanning. Increasing the pressure on the hydrophilized tip thus causes just nanobubble compression without tip penetration into the nanobubble volume.^31^ Note that the dull tip may limit the resolution of imaging.

Probes VTESPA-300 (cantilever resonant frequency *f* = 300 kHz, spring constant *k* = 42 N/m, nominal radius 5 nm, Bruker) and DNP-C (cantilever resonant frequency *f* = 56 kHz, spring constant *k* = 0.24 N/m, nominal radius 20 nm, Bruker) were utilized for scanning *ex situ* in tapping mode (TM) and PFQNM, respectively. Gwyddion (scanning probe microscopy data visualization and analysis software, version 2.58) was utilized for AFM image data processing.

All AFM images have axial (parameter axis) shade-coded with the gray scale corresponding to the scale of plotted parameter, i.e., the darker the color of the imaged region, the lower the parameter value. It should be noted that nanomechanical parameters obtained by PFQNM scanning presented in this paper do not have an absolute meaning and serve just for comparative purposes representing values related to nanomechanical properties of the rest of the scanned surface or neighboring surface area, as specified in captions to figures. Differences between the nanomechanical properties of gaseous nanodomains and the rest of the (wetted) basal plane surface are illustrated by spatial density distribution functions of reduced Young's modulus (DMT model) and adhesion to the hydrophilized AFM tip in Figure S1E,F, respectively.

Deionized water (shortened in text as DIW, purified by the Milli-Q system, Gradient, Millipore) with a resistivity of 18.2 MΩ·cm, equilibrated with air at 20 °C (oxygen concentration ∼10^–3^ mol·L^–1^), was used in the study. Alternatively, deionized water predegassed by combined procedures of water boiling at atmospheric pressure and cooling under reduced pressure, removing about 70–75% of dissolved ambient gas, was used. The glass cylinder with DIW preboiled at atmospheric pressure was closed, cooled down to 50 °C, and further cooled down to room temperature (20 °C) in the chamber at reduced pressure (∼2 kPa) until additional releasing of bubbles stopped. The cylinder was then sealed to avoid any residual gas volume left above water and used as a storage of degassed water for the experiment. The glass pipet was used for casting predegassed water on aged basal plane HOPG, which was then immediately engaged by the AFM head and scanned in PFQNM mode. Teflon or glass dish and glass pipettes served for water handling in all experiments.

The HOPG sample (Grade ZYB, size 12 × 12 mm^2^, Bruker, USA) freshly stripped by Scotch adhesive tape was placed in a glass Petri dish, closed to avoid accidental contamination by airborne dust and aerosol and stored for the time intended for sample exposure to an ambient atmosphere (i.e., “aging”) ranging from hours to several days. After the exposing time expired, the sample was immediately immersed in deionized water and its surface scanned *in situ* by AFM. Each experiment was repeated at least 2 times, and scanning was performed on three different surface locations. The surface from a previous experiment was renewed by stripping the top layers. Reference samples were always freshly stripped and immersed immediately after stripping in deionized water and scanned by AFM. To ensure the origin equality of both the surface used for aging and the surface used as a reference (fresh), the microtrench was cut on the basal plane HOPG, dividing its surface into two equal halves. After exposure to an ambient atmosphere, one-half of the basal plane surface was peeled off, serving as a reference surface, the whole sample was immediately immersed in water (DIW), and *in situ* AFM imaging was performed on both parts. A stable drop was formed on the basal plane, allowing us to perform *in situ* AFM (PF-QNM) scanning without utilization of liquid cell and O-ring sealing.

## Results and Discussion

**Figure 1:**
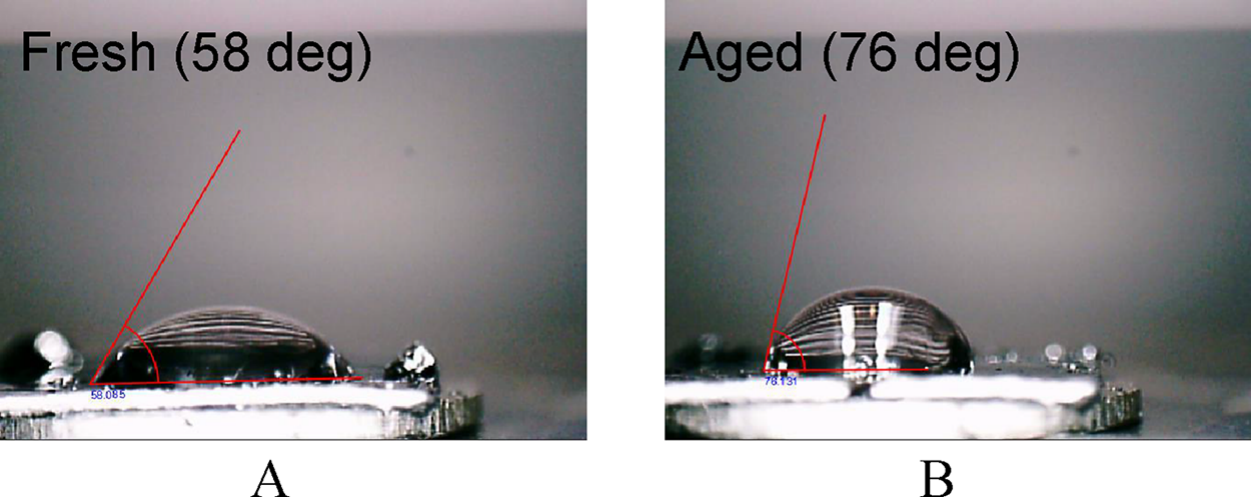
Difference in static contact angle at the ternary interface of a DIW drop on basal plane HOPG freshly peeled off (A) and aged for 92 h by exposing to an ambient atmosphere (B).

A water (DIW) drop placed on basal plane HOPG shows a significant difference in static contact angle between fresh and aged basal plane samples (measured from the water phase) and confirms earlier studies,^8^ indicating the difference in hydrophilicity of both surfaces. The so-called aging causes a shift of surface properties toward hydrophobicity by increasing the water contact angle, as shown in Figure 1.

**Figure 2:**
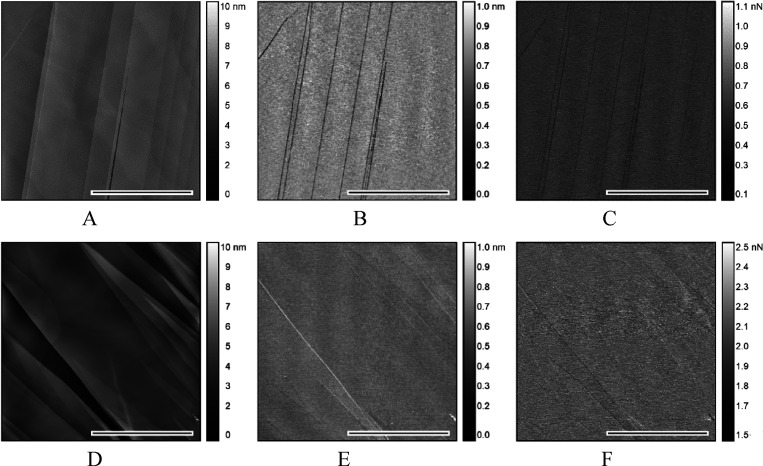
Typical *ex situ* AFM PeakForce QNM images of basal plane HOPG freshly peeled (A–C) and aged for 50 h by exposing to an ambient atmosphere (air) (D–F). The black-white bar represents the image lateral scale of 5 μm. (A) and (D) present the height images, (B) and (E) the deformation, and (C) and (F) the adhesion images.

On the other hand, no significant difference of surface nanomorphology and nanomechanical properties (PeakForce QNM imaging) was found for aged basal plane HOPG compared to the freshly stripped surface if examined *ex situ* (dry) (Figure 2).

**Figure 3:**
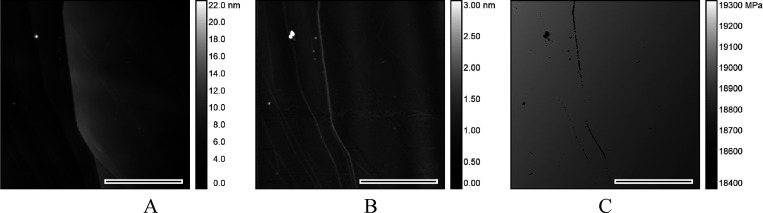
*In situ* AFM height (A), PFQNM deformation (B), and reduced Young's modulus (DMT model) (C) images of freshly peeled-off basal plane HOPG immersed in DIW. The black-white bar represents the image lateral scale of 2 μm.

Immersion in DIW however makes a remarkable difference between fresh and aged HOPG as revealed by *in situ* PFQNM imaging: freshly stripped basal plane HOPG immersed in water shows its typical nanomorphology without any significant irregularities of nanomechanical parameters (Figure 3) compared to *ex situ* images (Figure 2).

**Figure 4:**
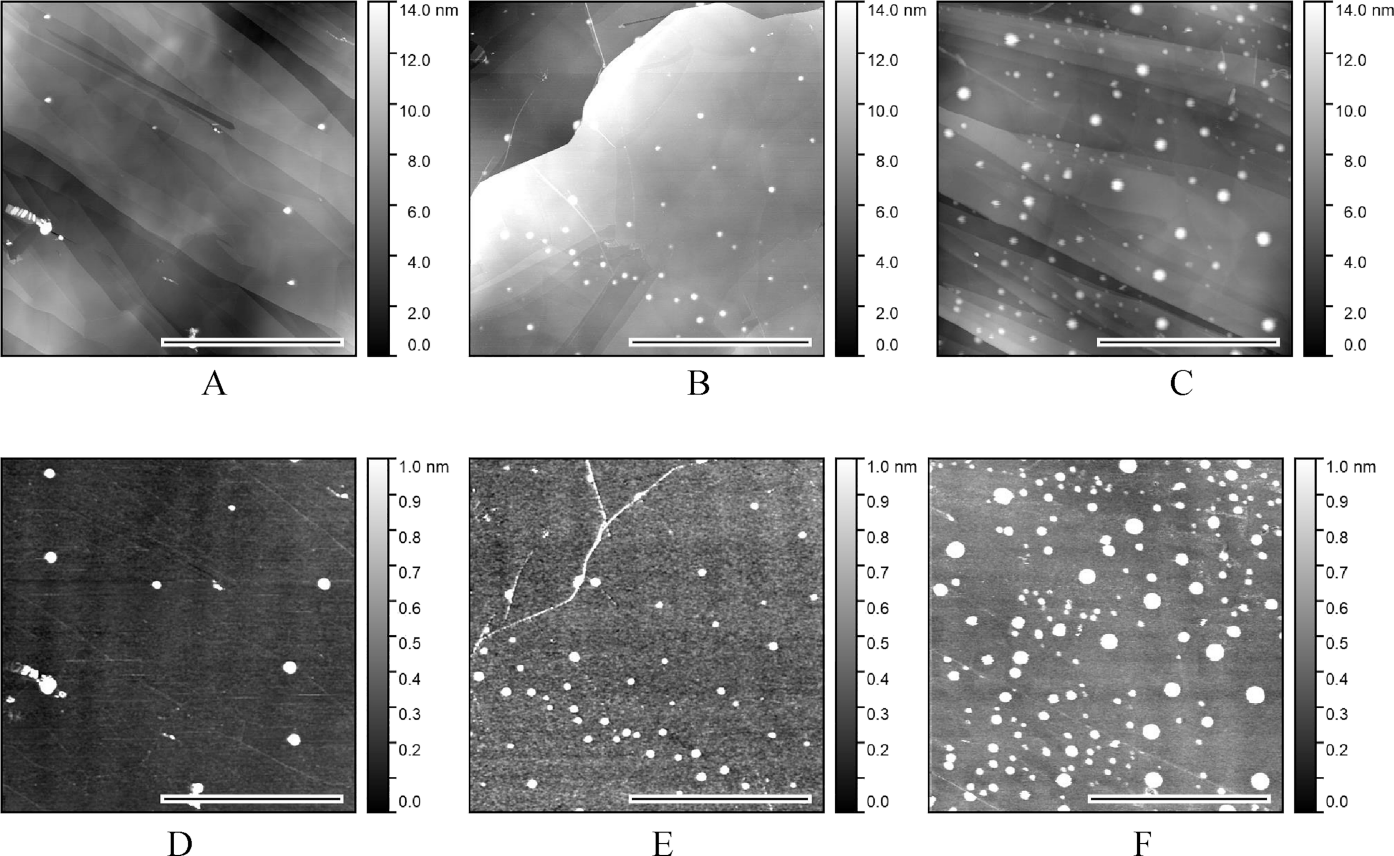
Typical *in situ* AFM height (A–C) and PFQNM deformation (D–F) images of surface ambient gaseous nanobubbles formed spontaneously on water-immersed basal plane HOPG, exposed before immersion to an ambient atmosphere (air, 20 °C) for 2, 23, and 96 h, respectively. The black-white bar corresponds to 5 μm on the lateral scale.

On the contrary, aged basal plane HOPG immersed in DIW is populated by surface nanobubbles, characterized by higher elastic deformation values compared to the rest of the HOPG surface (Figure 4D–F). Elastic compression by increasing the pressure on the tip indicated decreasing both lateral (surface diameter) and axial (apex height) dimensions without shape deformation, which are properties characteristic for nanobubbles.^32^ High stability upon repeated scanning is characteristic for strong nanobubble pinning.^28,33,34^ The negative phase shift of tapping mode (TM) phase imaging confirmed the elastic stiffness of gaseous nanodomains, while the conditions of negligible adhesive forces^35^ between the tip and gaseous surface were met for the hydrophilized AFM tip. The TM with a negative phase shift and PFQNM showing low *Y*_M_(DMT), high deformation, and low adhesion to the hydrophilic tip are presented in Figure S1 (Supporting Information). The topography height images of surface nanobubbles, scanned by AFM at different applied forces (setpoints 0.9 and 1.5 nN), clearly show symmetrical compression (Figure S2 in the Supporting Information). Similarly, nanobubbles scanned in deformation (PFQNM) mode, the steadily increasing force at the rising part of the force curve imposed on nanobubbles, exhibited symmetrical deformation, as shown by two orthogonal profiles drawn across each nanobubble deformation image (Figure S3 in the Supporting Information), implying their gaseous origin.

Nanobubble incidence was found to rise significantly with prolonged surface exposure to an ambient atmosphere (aging) as illustrated by the AFM images of surfaces aged for 2, 23, and 96 h (Figure 4). A similar trend was observed by Hu et al.,^30^ explained by changes of the nanobubble nucleation rate on the hydrophobic surface.

Differences between freshly prepared and aged basal plane HOPG observed upon immersion in water can be ascribed to different hydrophilicities: while the freshly stripped HOPG surface exhibits rather hydrophilic properties and thus higher attractivity to an aqueous phase (higher wettability), the aged surface has significant hydrophobic properties^36^ ascribed to surface adlayers, reportedly formed by deposition of airborne contaminants,^8^ and represents thus a surface less susceptible to gas displacement by aqueous media.

Our explanation is based on the water repelling behavior of the hydrophobic surface, which causes its incomplete wetting during immersion, and ambient gas is due to preferable adherence to the hydrophobic surface dragged into water, where it forms surface gaseous nanodomains. This concept of incomplete wetting already addressed by Simonsen et al.^37^ is also supported by the immediate appearance of surface gaseous nanodomains after surface immersion in water, while nanobubble nucleation is a time-consuming process.^38^ As the direct source of gas for incomplete wetting is the ambient gas phase (atmosphere), not the gas dissolved in water, the process is independent of gas concentration in the water phase. Accordingly, the AFM (PFQNM mode) image of the aged surface of basal plane HOPG immersed in predegassed water shows surface gaseous nanodomains with incidence similar to water equilibrated with air as illustrated by Figure S4 in the Supporting Information. This finding, in accordance with the literature^26,39^ reporting the appearance of gaseous nanodomains in partially degassed water, supports the incomplete wetting concept. To further clarify this point, we have performed experiments utilizing total internal reflection (TIR) of incident light for identification of surface gaseous domains formed during immersion of the model carbonaceous hydrophobic surface in degassed water, which confirmed the incomplete wetting (ambient gas dragging) mechanism of formation gaseous microdomains upon immersion in degassed water presented in Figure S5 (Supporting Information).

The remarkable difference between the AFM images of the dry aged HOPG surface and after its immersion in water can be explained by the modification of the basal plane surface by airborne contaminants, forming an ultrathin subnanometer film, which is hardly recognizable by *ex situ* AFM.^30^ Its immersion in water leads to trapping of ambient gas on hydrophobized (aged) HOPG creating gaseous nanodomains with dimensions significantly exceeding the nanometer scale and thus easily visualized by *in situ* AFM imaging.

**Figure 5:**
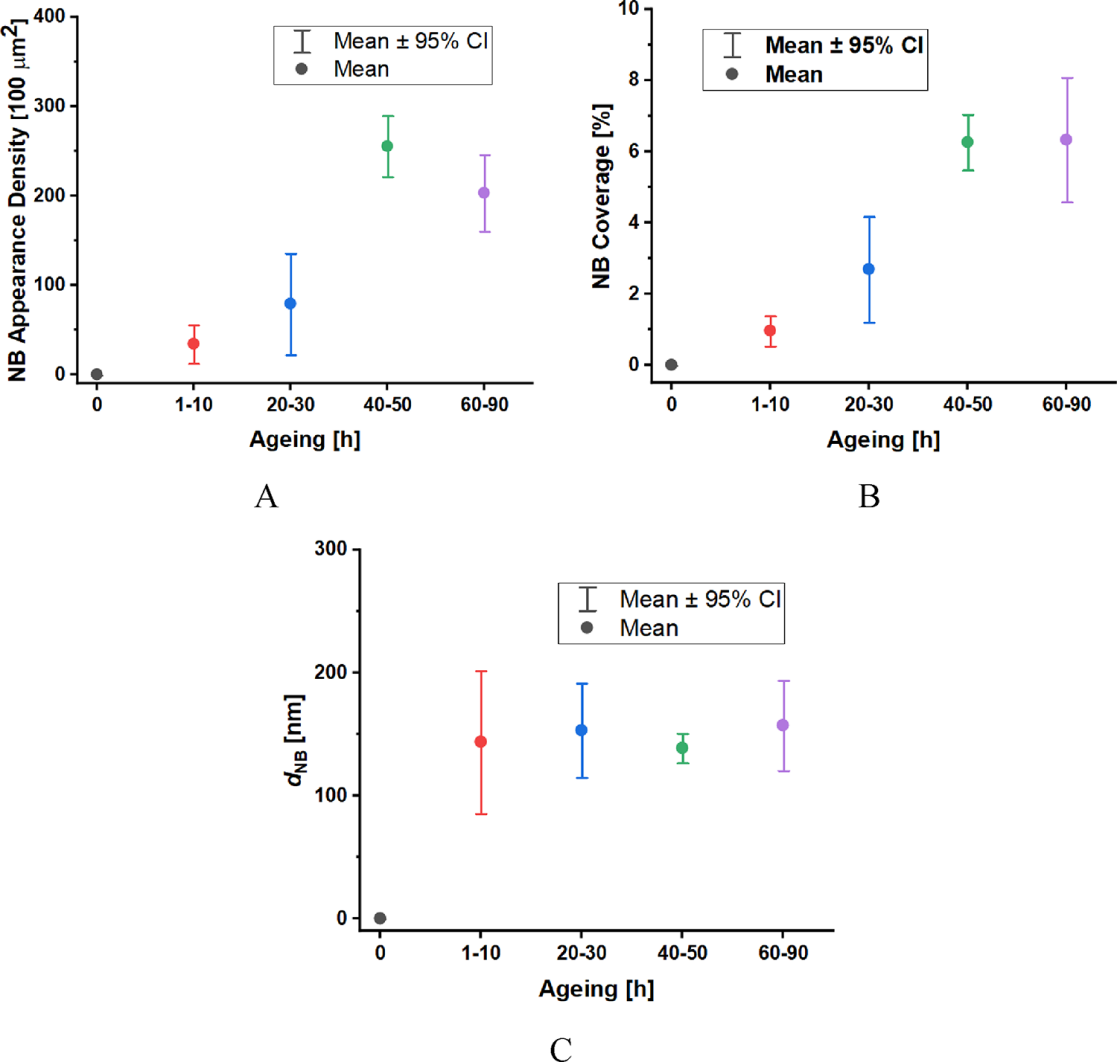
Statistics of surface nanobubble population on basal plane HOPG immersed in deionized water in relation to HOPG aging time (in h): appearance density (A), surface coverage (B), and nanobubble diameter (*d*_NB_) (C). Error bars show the spread from the mean value within 95% confidence interval. The nanobubble diameter corresponds to the nanobubble footprint diameter and the term appearance density represents the number of gaseous nanodomains appearing in the defined area of the imaged surface (100 μm^2^).

Prolonged aging of the HOPG surface by exposure to an ambient atmosphere (air) causes an increase in nanobubble population on the water-immersed HOPG surface, as summarized by statistic plots in Figure 5A, B.

The nanobubble appearance density rises with HOPG aging time and reaches the limit on surfaces aged for 40–50 h, while the surface nanobubble diameter is steadily distributed between 100 and 200 nm (Figure 5C), resembling the diameter prevalence found for bulk nanobubbles^40,41^ with no significant dependence on surface aging. Certain data fluctuation can be ascribed to the spread of surface properties among different layers obtained by each exfoliation, differences among samples examined in experiments,^42^ and possible day-to-day variation of airborne contaminants.^8^

On the other hand, water immersion appears to protect the surface of freshly stripped basal plane HOPG from “aging”. Our finding, in accordance with Li et al.,^43^ confirms the widely accepted concept that the contamination is solely airborne,^4^ while Hu et al.^30^ claimed that aging proceeds in water as well.

**Figure 6:**
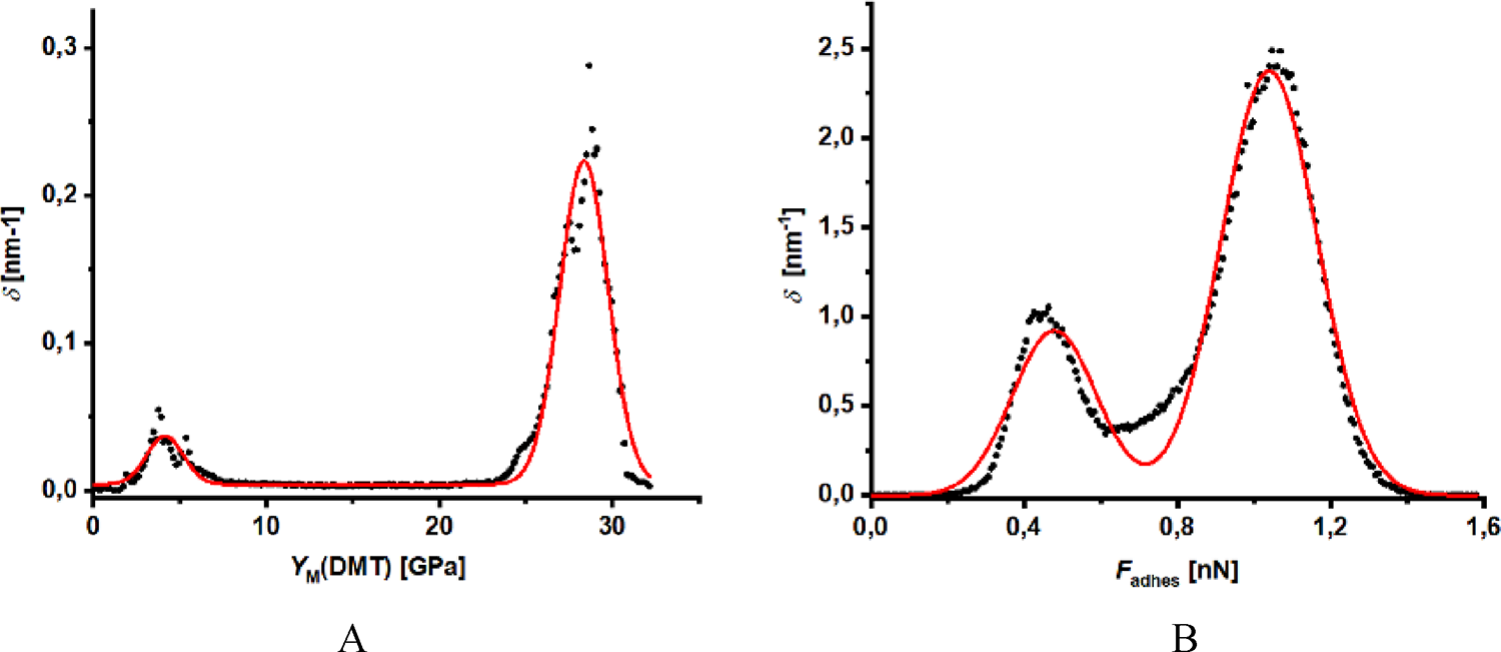
Spatial density distribution functions distinguish nanomechanical properties reduced Young's modulus (DMT model) (A) and adhesion to the hydrophilized AFM tip (B) of gaseous nanodomains (first peak) from the rest of the wetted HOPG surface (second peak). Red curves represent the best fits (Gauss).

The spatial density distribution functions of nanomechanical parameters, Young's modulus (DMT), and adhesion, acquired from the PFQNM mapping of the water immersed surface, illustrate the gap between the nanomechanical parameters of gaseous nanodomains and the bare wetted surface (Figure 6).

**Figure 7:**
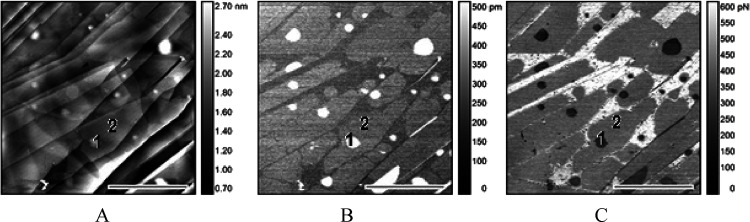
Typical *in situ* AFM height (A) and PFQNM (B: deformation, C: adhesion) images of aged (47 h) basal plane HOPG immersed in deionized water (20 °C). Images show the surface populated by ambient gaseous nanodomains: nanobubbles (1) coexisting with micropancakes (2). The black-white bar represents the image lateral scale 2 μm.

A further increase in aging time causes, besides population of circularly shaped nanobubbles (marked 1 in Figure 7), formation of micropatches of irregular shapes (marked 2 in Figure 7). The PeakForce QNM imaging reveals their low and uniform thickness (∼1 nm), higher deformability, and lower adhesion to the hydrophilized AFM tip (Figure 7B,C) compared to the rest of the HOPG surface, which can indicate the presence of ultrathin gaseous layers—micropancakes,^23^ considered in some works to be a form of surface gas condensate^14,44,45^ or interfacial gas enrichments.^46,47^

In comparison with surface nanobubbles, the deformability of micropancakes is somewhat lower (Figure 7B 1,2), as noticed also by Zhang et al.^23^ It can be ascribed to the lower thickness and therefore lower deformation height compared to more voluminous nanobubbles, the apex of which is regularly an order of magnitude higher. This difference is particularly evident for nanobubble–micropancake composites, where nanobubbles coexist with micropancakes^23^ (Figure 7 and Figure S1A,B).

**Figure 8:**
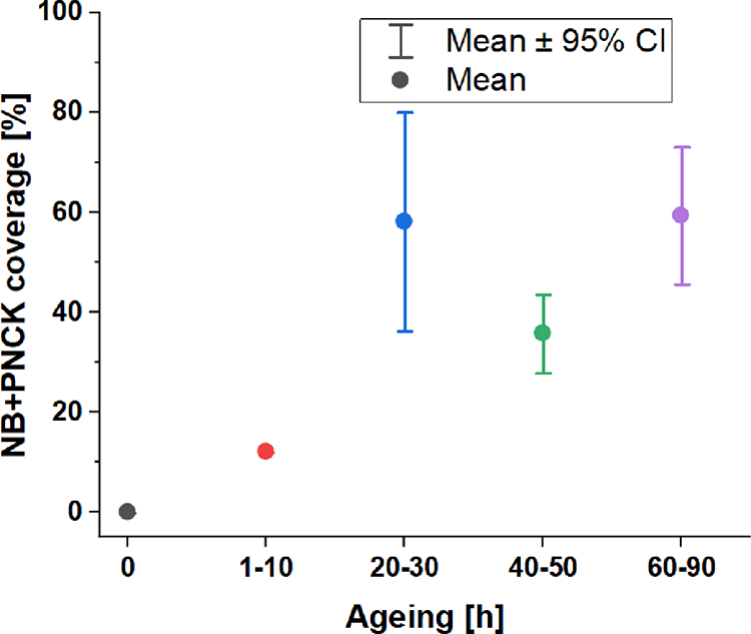
Statistic of surface coverage by gaseous nanodomains (both nanobubbles and micropancakes) populating basal plane HOPG upon its immersion in deionized water, in relation to HOPG aging time (in h). Error bars correspond to the spread from the mean value within a 95% confidence interval.

Similarly, as for nanobubbles, the appearance density of micropancakes on water-immersed basal plane HOPG rises with graphite aging: micropancakes cover up to about 60% of the immersed surface once aging times exceed 24 h (Figure 8), while on freshly peeled HOPG surfaces as well as on surfaces below an aging time of 10h, no micro/nanopancake was identified.

Following from statistic plots (Figures 5 and 8), the extent of surface coverage by gaseous nanodomains, both by micropancakes and nanobubbles formed on the aged graphite surface just upon its immersion, represents a barrier, which may cause significant hindering of interfacial processes: In electrochemistry, the direct relation exists between the extent of electrochemical reaction (expressed as electrochemical current) and the surface area, which is in contact with the liquid phase (electrolyte solution), i.e., wetted surface area. (see, e.g., ref (48)). It indicates that any variation of wetted surface area causes variation of the reaction extent, reflected by the magnitude of electrochemical current. Because it is known that micro- and millibubbles on the electrode surface can deteriorate its electrochemical performance^49^ by shielding the electrode surface from direct contact with electrolyte solution, we may expect a similar effect caused by nanobubbles, which can populate large parts of the immersed hydrophobic surfaces. Gaseous nanodomains covering up to 60% of the immersed HOPG surface restrict thus significantly its contact with the liquid phase, which may explain so far the unclear observation of strong correlation between electrochemical rate constants and HOPG aging, exhibited by slowing down reaction kinetic^16−18^ and declining capacitances.^9^ It should be noted that, several years ago, Spuller and Hess^50^ already pointed out the importance of immersion as the first step of all interfacial processes, which are conditioned by the quality of solid–liquid contact, i.e., by wetting. They stated the general neglection of this first step by the widely accepted assumption of complete surface wetting, which, however, becomes invalid whenever wetting is obstructed. The wetting process starts with the dry solid surface initially in contact with ambient gas, which must be displaced fully by the liquid upon immersion. Our finding that exposure of the graphite (HOPG) surface to an ambient atmosphere leads to a significant reduction of wetted surface by gaseous nanodomains contributes to so far the opened question of how graphite aging influences its utilizations as a material for electrochemistry and heterogeneous catalysis.^2^

## Conclusions

In this work, we have shown differences between the water-immersed freshly prepared HOPG surface, which is fully wetted, and the aged surface, where gaseous nanodomains block its significant (up to 60%) part. As the partial degassing does not significantly affect the appearance of surface gaseous nanodomains, together with the fact that surface nanobubbles appear immediately after immersion, we can assume that ambient gas dragging (i.e., incomplete wetting) caused formation of gaseous nanodomains even at a low gas concentration in water, which implies difficulty in preventing their formation upon immersion of hydrophobic surfaces.

Continuation of our work will be focused on clarification of the mechanism by which surface gaseous nanodomains affect the kinetic parameters of charge and mass transfer processes at the solid–liquid interface.

## Supporting Information Available

Nanomechanical properties of gaseous nanodomains; compression of nanobubbles; deformation of nanobubbles; gaseous nanodomains in predegassed water; identification of incomplete wetting by total internal reflection (PDF)
