# Supplementary material for: Effect of Graphite Aging on Its Wetting Properties and Surface Blocking by Gaseous Nanodomains
Source: Langmuir. 2023 Sep 21;39(39):14154–61. doi: 10.1021/acs.langmuir.3c02151 (PMC10552534; doi:10.1021/acs.langmuir.3c02151)
Supplement: Supplementary file 1 — la3c02151_si_001.pdf [file la3c02151_si_001.pdf]

Supporting Information

Effect of graphite ageing on its wetting properties and surface blocking by gaseous nanodomains

*Hana Tarábková, Pavel Janda**

Department of Electrochemical Materials, J. Heyrovský Institute of Physical Chemistry, Czech Academy of Sciences, Dolejškova 2155/3, CZ-182 23 Prague 8, Czech Republic

Corresponding Author

* E-mail: pavel.janda@jh-inst.cas.cz

Table of Contents

1. Nanomechanical properties of gaseous nanodomains
2. Compression of nanobubbles
3. Deformation of nanobubbles

4. Gaseous nanodomains in pre-degassed water

5. Identification of incomplete wetting by Total Internal Reflection

1. Nanomechanical properties of gaseous nanodomains

Surface gaseous nanodomains are identified by both nanomechanical and surface properties, indicated by negative phase shift, low Y_M(DMT), high deformation and low adhesion to hydrophilic tip, which significantly differ from the rest of (wetted) surface, as presented in Fig. S1 and Fig. 6.

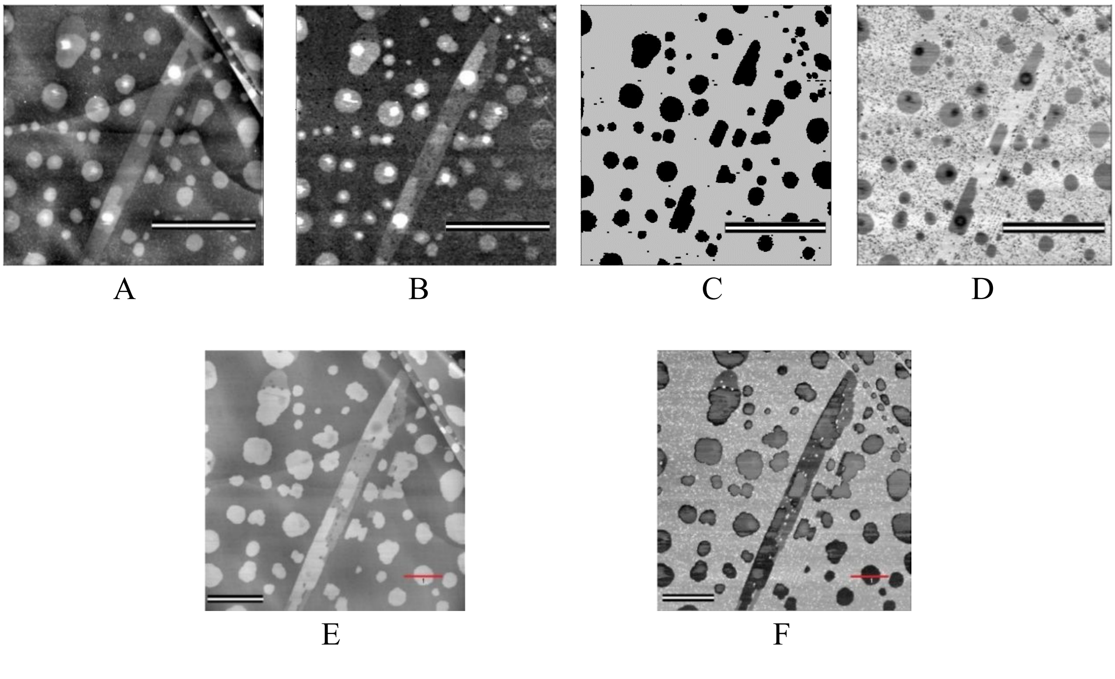

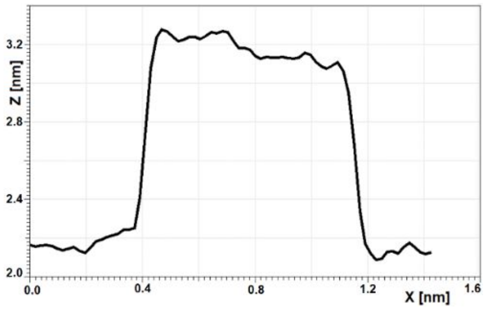

G

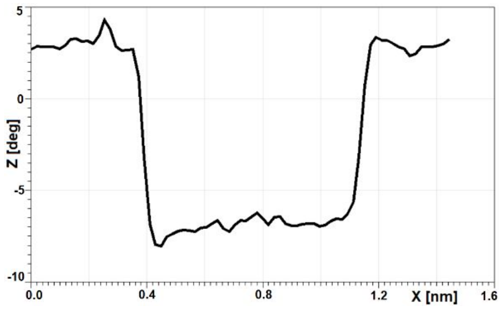

H

Figure S1: The in-situ PFQNM (A-D) and TM (G, H) images of identical location of basal plane HOPG, aged for 48h: Height (A), deformation (B), reduced Young Modulus (DMT model) (C), adhesion (D) to hydrophilized AFM tip. Profiles (G, H) illustrate the relation between TM height (G) and negative phase shift (H) of gaseous nanodomain from Figs E, F. The location of profile line is marked by red lines (1) in both Figs E and F. Scales: The maximum axial (z) scale range is 1.5 nm for PFQNM height (A) and deformation image (B), 25 GPa for Young modulus (DMT) image (C) and 1.3 nN for adhesion image (D). For tapping images, the maximum axial (z) scale is 4 nm for height image (E) and 15° for phase shift image (F). The black-white bar represents the lateral scale 4 μm for images A-D and 2 μm for images E, F.

As follows from Fig. S1F, the highest contrast in tapping phase images corresponds to areas identified by PFQNM analyses (DMT and deformation) as “soft” (Fig. S1B, C), assigned to gaseous nanodomains, where also the adhesion to hydrophilized tip is significantly lower, compared to rest of (wetted) solid surface (Fig. S1D). Though all values of nanomechanical

parameters just illustrate their difference between graphite and gaseous surface and do not represent absolute values, YM fits well to the range known for HOPG surfaces 1,2.

2. Compression of nanobubbles

Different forces (setpoints 0.9 nN and 1.5 nN) applied on the nanobubbles by the AFM tip, caused nanobubble symmetrical compression leading to the change of its dimensions – the decrease of apex height, as shown in Fig. S2. The dimension change depends, besides applied pressure, also on nanobubble pinning to the solid surface.

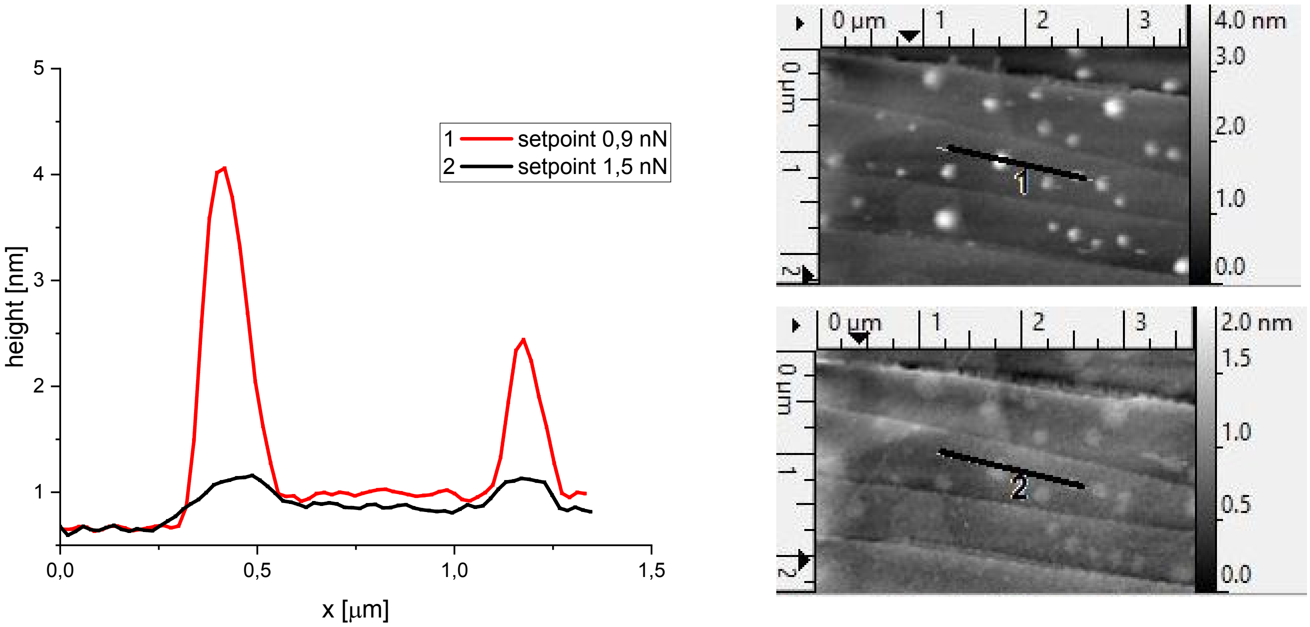

Figure S2: Profile lines (left) across gaseous nanobubbles formed on aged (45h) basal plane HOPG immersed in deionized water. Nanobubble height images at identical location (right) were obtained by in-situ AFM scanning (PFQNM mode) at peak force setpoints 0.9 nN (profile 1) and 1.5 nN (profile 2).

3. Deformation of nanobubbles

When surface nanobubbles are scanned in deformation (PFQNM) mode, the steadily increasing force at rising part of the force curve imposed on nanobubbles causes their symmetrical deformation without any anomality, as shown by two orthogonal profiles drawn across center of each nanobubble deformation image (Fig. S3).

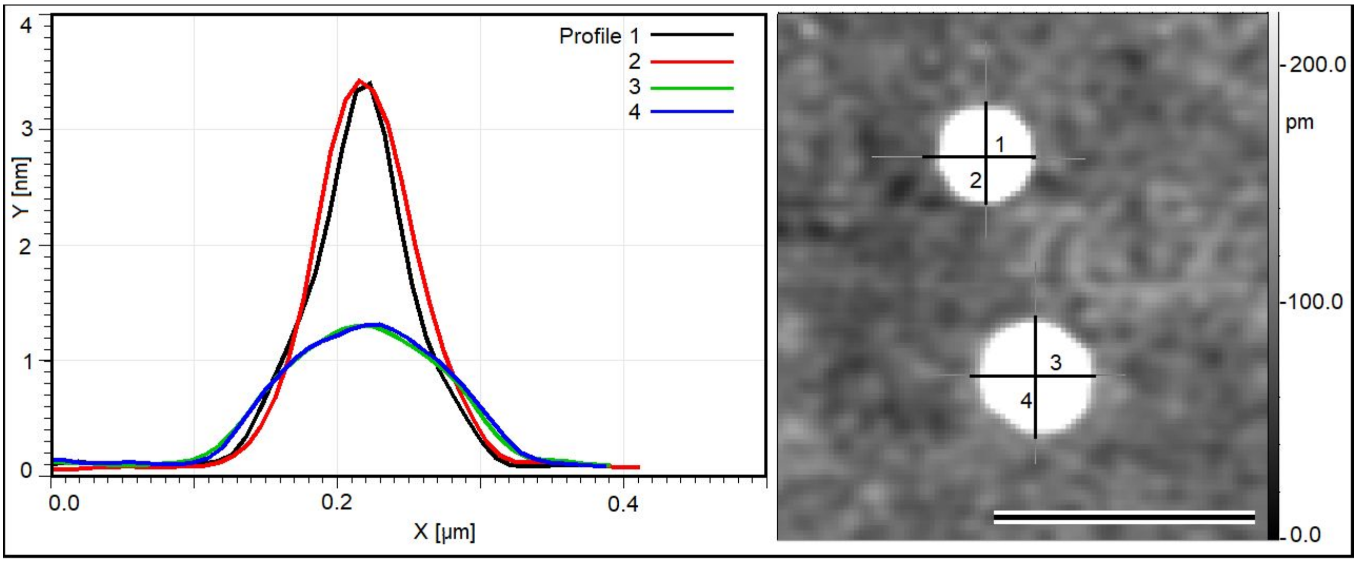

Figure S3: Profile lines (left) drawn along two orthogonal axes (1,2; 3,4) across apex of each gaseous nanobubble formed on aged (25h) basal plane HOPG surface immersed in pre-degassed

water. Nanobubble imaging was performed by in-situ AFM PFQNM in deformation mode at peak force setpoint 0.5 nN.

4. Gaseous nanodomains in pre-degassed water

AFM (PFQNM) images of basal plane HOPG aged for 25 h, immersed in pre-degassed deionized water (DIW) show both nanobubbles and micropancakes - appearing just upon immersion (Fig. S4). Their incidence in pre-degassed water, where gas concentration was lowered, is similar to water equilibrated with air, which indicates, that partial degassing does not significantly affect incidence of surface gaseous nanodomains formed upon immersion. This finding thus supports participation of incomplete wetting in surface nanodomain formation upon immersion.

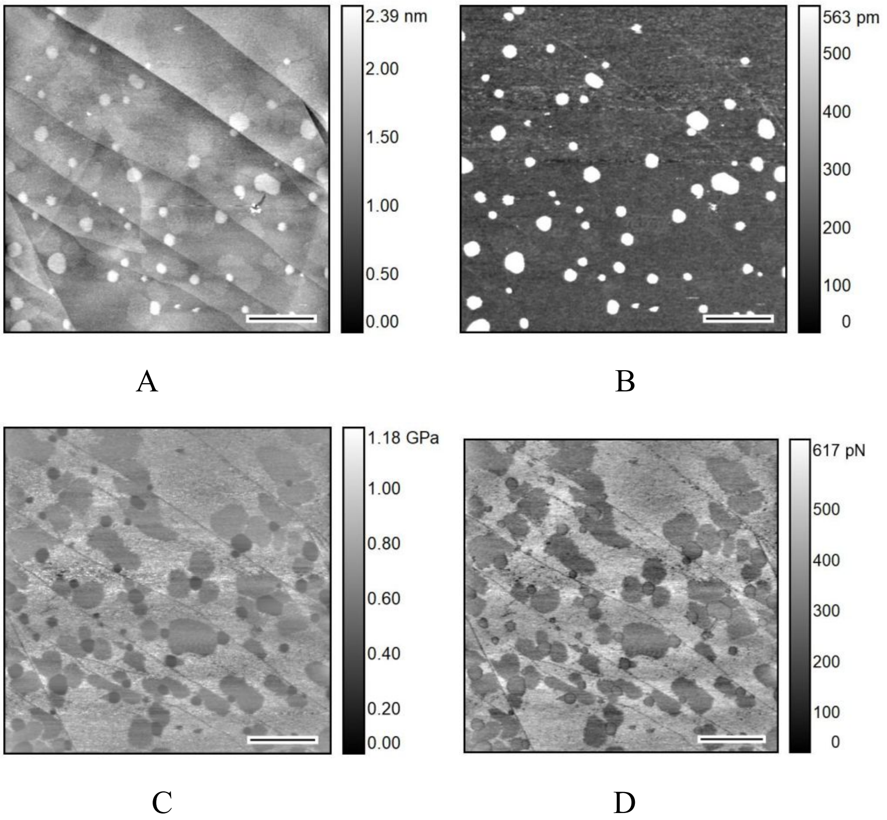

Figure S4: The in-situ AFM (PFQNM) images - height (A), deformation (B), reduced Young Modulus-DMT/stiffness (C) and adhesion to hydrophilized tip (D) of 25h-aged basal plane HOPG immersed in pre-degassed water. Scale bars correspond to 1 μm, peak force applied on the tip (setpoint) was set to 0.5 nN.

5. Identification of incomplete wetting by Total Internal Reflection

To further clarify the participation of incomplete wetting on the formation of surface gaseous domains, modified experiment utilizing total internal reflection (TIR) of incident light is presented for identification of gaseous microlayers formed on hydrophobic surfaces upon immersion in water: Silicon wafers with flame-deposited carbon (FDC) mimicking

hydrophobic surface of aged HOPG were utilized as model samples with carbonaceous hydrophobic surface allowing direct optical visualization of surface gaseous layers by TIR. Samples were immersed in water equilibrated with air at 20ºC and in water pre-degassed by boiling, which was kept heated to 87ºC to minimize re-gassing. In both cases gaseous microlayers formed on FDC surface are clearly visible by manifesting TIR on mirror-like gas-liquid interface[3], as shown in Fig. S5. This experiment is thus proving feasibility of ambient gas dragging (incomplete wetting) to create surface gaseous domains just upon immersion, independently on gas concentration in water. It should be noted, that visualization of TIR effect is possible if thickness of gaseous layer exceeds the penetration depth of evanescent wave, which is in orders of 10^2 nm. Therefore, the direct visual detection of gaseous nanodomains by TIR is not possible, due to their thickness typically below 10 nm.

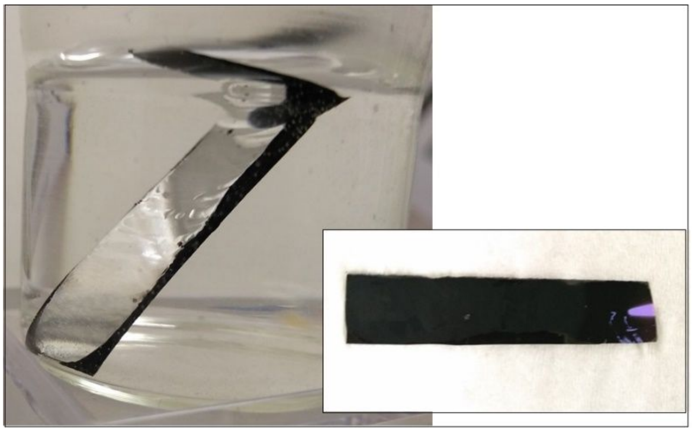

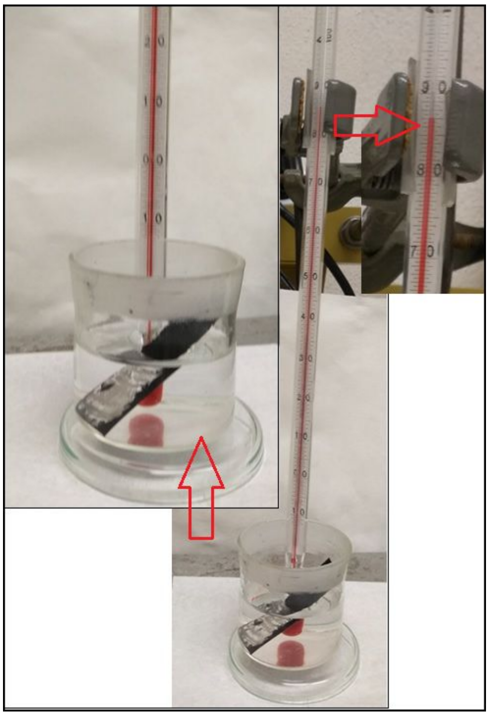

Figure S5: Silicon wafer coated with flame-deposited carbon (FDC, insert in left image) is immersed in deionized water equilibrated with ambient atmosphere (air) at 20°C (left) and in water pre-degassed by boiling at atmospheric pressure; immersion at temperature 87°C (see thermometer immersed in bath and zoomed thermometer scale, right). Red arrows point to zoomed images

REFERENCES

(1) Song, Y. S.; Qi, L. H.; Li, Y. X. Prediction of Elastic Properties of Pyrolytic Carbon Based on Orientation Angle. IOP Conf. Ser. Mater. Sci. Eng. 2017, 213 (1), 012030. https://doi.org/10.1088/1757-899X/213/1/012030.

(2) Abooalizadeh, Z.; Sudak, L. J.; Egberts, P. Nanoscale Spatial Mapping of Mechanical Properties through Dynamic Atomic Force Microscopy. Beilstein J. Nanotechnol. 2019, 10, 1332–1347. https://doi.org/10.3762/BJNANO.10.132.

(3) Hoang, C. H.; Nguyen, T. T.; Ho, D. Q.; Le, H. V.; Nguyen, H. H. Fabrication of Superhydrophobic Surfaces for Applications in Total Internal Reflection Effects. Mater. Today Commun. 2023, 35 (April), 105928. https://doi.org/10.1016/j.mtcomm.2023.105928.
